# How do locus of control influence business and personal success? The mediating effects of entrepreneurial competency

**DOI:** 10.3389/fpsyg.2022.958911

**Published:** 2023-01-05

**Authors:** Muhammad Iskandar Hamzah, Abdul Kadir Othman

**Affiliations:** ^1^Faculty of Business and Management, Universiti Teknologi MARA Puncak Alam, Selangor, Malaysia; ^2^Institute of Business Excellence, Universiti Teknologi MARA Shah Alam, Selangor, Malaysia

**Keywords:** entrepreneurial competency, locus of control, business growth, quality of life, sustainable entrepreneurial intention, innovative, analytical, opportunity-seeking

## Abstract

This research aims to assess the influence of locus of control on the expression of entrepreneurial competency in a small business setting. Specifically, it predicts how this can generate positive outcomes in terms of business growth, quality of life, and sustainable entrepreneurial intention. Survey responses were collected from 102 small-sized firms in Malaysia. Structural equation modeling was performed to validate a mediation model and test nine research hypotheses. The results suggested that internal locus of control indirectly affects the venturing outcomes *via* entrepreneurial competency, whereas external locus of control has no such consequences. Thus, it can be deduced that beliefs based on internal attributions—rather than external forces, define entrepreneurs’ destiny, and their competencies serve a perpetual role in linking these beliefs to positive business performance, life satisfaction, and sustainable entrepreneurial behavior. In practical terms, policymakers may gradually shift their focus from supplying direct financial relief assistance to the owner-managers to empowering them with core competencies building programs, especially during disasters and recessions. This study unravels the complexities of the entrepreneurial psychology-competency interface and fills a gap in the literature by providing compelling evidence of the adverse consequences of relying too heavily on fate or external assistance.

## Introduction

The study of entrepreneurial competency has often led to conflicting views about what motivates such ability and the outcomes, typically measured *via* entrepreneurial success. According to the entrepreneurship literature, entrepreneurial success is generally agreed to be influenced by intrinsic individual and extrinsic environment elements. However, little is understood about how entrepreneurs might capitalize on entrepreneurial prospects *via* this route. The potential answer may arise from their enterprising qualities, which may explain why some individuals behave differently than others in the same situation. The entrepreneurial competency framework should accurately reflect these characteristics (Bird, 1995). Against this backdrop, academics have argued that the growth and development of nomological networks interacting with this construct remain comparatively slow and obscure. This ambiguity is exacerbated by the fact that entrepreneurial competencies are researched in numerous countries and cultural settings.

In his extensive meta-analytic review, Jain (2011) emphasized that, eventhough one’s perceived personal control of business outcomes is more internally oriented for entrepreneurs than non-entrepreneurs, more genuine empirical observations are needed to build more comprehensive frameworks involving entrepreneurial competency and locus of control. The ongoing theoretical debate on entrepreneurs’ psychological dispositions and personalities, particularly amid adversity, appears unabated. Several scholars, for instance, have recommended the incorporation of locus of control as one of the most important aspects of entrepreneurial competency (Jain, 2011; Lee et al., 2016). However, entrepreneurial ability and skills are dynamic in nature (Mitchelmore and Rowley, 2010; Tittel and Terzidis, 2020), whereas locus of control is constant over time due to the greater influence of cultural, social, and terminal values (Hartmann et al., 2022). Thus, we emphasize the urgent need for further theoretical clarification to increase the value of empirical contributions pertaining to entrepreneurial competency’s antecedents and outcomes.

Generally, across the society, income generation is often thought to serve as the primary indicator of entrepreneurial success. Success certainly appears tempting to aspiring entrepreneurs when measured in economic and monetary terms. However, economic success from building wealth does not guarantee satisfaction with one’s psychological wellbeing with his or her commercial endeavor. Existing studies have attributed entrepreneurial competency to tangible outcomes, namely business performance and wealth generation (see Mitchelmore and Rowley, 2010; Al Mamun and Fazal, 2018; Reis et al., 2020). Yet, entrepreneurial competency has rarely been investigated at the psychological interface, specifically by relating it to intrinsic and intangible consequences.

This materialistic view contradicts the general motivation principle that higher-order intrinsic rewards (e.g., livelihood improvement and ideal self) matter more than lower-order tangible rewards (e.g., income) (Shiferaw, 2020). Consequently, this issue offers us the opportunity to narrow the knowledge void by exploring the connections among entrepreneurial traits, entrepreneurial competency, and intrinsic outcomes. Notably, there is a lack of a distinctive model that links this competency with psychological wellbeing, entrepreneurial sustainability behavior, and their locus of control. There are several questions that were left unanswered. For instance, does competency lead to personal contentment with life and business continuity, and to what extent does entrepreneurs’ sense of self-control assist them in achieving these outcomes? To subdue this confusion, we offer a framework that predicts a holistic entrepreneurial competency outcome based on business growth, quality of life, and sustainable entrepreneurial intention. Subsequently, these effects are proposed to be indirectly predicted by the entrepreneurs’ locus of control.

The contribution of this study is 2-fold. First, this work tests a model that investigates the nature of the relationship between business growth, quality of life, sustainable entrepreneurial intention, entrepreneurial competency, and internal and external locus of control. To reiterate, the use of tangible (business performance) and intangible outcomes (quality of life and sustainable entrepreneurial intention) partly contributes to this study’s novelty. Second, this study is among the earliest attempts to examine the mediating role of entrepreneurial competency on the relationship between locus of control dimensions and the outcome variables. The theoretical framework and hypotheses are presented in the subsequent section, followed by a discussion of the research methods and findings. The paper concludes with discussions on theoretical and managerial implications, limitations, and future research directions.

### Self-determination theory and entrepreneurial success

Entrepreneurial success has been widely covered across the entrepreneurial psychology literature stream (e.g., Mitchelmore and Rowley, 2010; Khan et al., 2021), but its conceptualization remains unclear. Entrepreneurial success manifests in many forms (e.g., financial performance and business growth), but in general, the extant research lacks analysis at the individual level. Entrepreneurs’ decisions to venture into business startups-while taking a risk to earn an uncertain living by leaving fixed-salary employment are motivated mainly by personal aspirations. These life goals typically revolve around attaining tangible rewards (economic values) and intangible rewards (self-actualization and self-esteem). The desire to improve one’s quality of life frequently serves as a strong driver to overcome the risks of starting a business (Peters and Schuckert, 2014).

According to the self-determination theory (SDT), three basic and universal psychological requirements propel people to grow and change namely autonomy, competence, and relatedness (Ryan and Deci, 1985). SDT’s application is relevant to the entrepreneurial field since entrepreneurs must be able to make decisions and govern their own lives to achieve a decent state of psychological wellbeing. Generating wealth alone would not suffice in representing success since people need to gain intrinsic rewards to appreciate and continue what they are pursuing. People’s inner strength is driven by psychological contentment, and it is this source of motivation that allows entrepreneurs to persevere in the face of hardship and endless challenges (Chakraborty et al., 2019).

Unlike bigger corporations run by teams of managers, small business entrepreneurs frequently make decisions without consulting other members of the organization, banking only on their own abilities, and experience (Man et al., 2002). Their self-determination and self-motivation propel them to be responsible for shaping their life destiny amid the uncertainty of generating a steady flow of income. Entrepreneurs are attracted to start a venture through time and money considerations, amid the common belief that fixed-income employment would not provide equivalent financial and non-pecuniary benefits. Contrary to salary earners’ fixed working hours and prescribed tasks, entrepreneurs can harness time flexibility for balancing work-life matters and unravel hidden potential for personal development. Besides materialistic gains, these benefits also provide the route for physical, emotional, mental, and social wellbeing goals. Thus, the desire to reach a higher quality of life increases naturally.

Therefore, we argue that psychological wellbeing should be considered alongside other achievement goals such as business growth and continuity when analyzing entrepreneurial success *via* the lens of the SDT paradigm.

### The entrepreneurial competency concept

For most small businesses captained by a single individual, personal differences or qualities act as determinants that explain how some entrepreneurs are more successful than others. Entrepreneurial competencies are defined as an individual’s underlying attributes that lead to the formation of new ventures. In 1995, Barbara Bird proposed one of the earliest entrepreneurial competency concepts built on work of Boyatzis (1982) on managerial competencies. Competence forms an integral part of an entrepreneur’s internal psychological state. It is more closely linked to venture performance than other psychological characteristics such as personality traits and internal motivation (Bird, 1995). In this regard, competencies act as enablers for behaviors of various entrepreneurial qualities, but they are not behaviors themselves.

The academic debate on this topic has centered on constructing a functional model of entrepreneurial competencies. In view of this concern, qualitative work of Bird (1995) was further validated into an empirical framework and a set of instruments that measures SME owner competitiveness *via* four dimensions: relational, innovativeness, analytical, and opportunity seeking (Man et al., 2008). Other than taxonomy of entrepreneurial competency of Bird (1995), the entrepreneurial competency concept is expressed across the literature through multiple knowledge streams. Many of these conceptualizations follow the knowledge-skills-attributes (or KSA) formula (Man et al., 2002).

For instance, interpretation of entrepreneurial competency of Cheetham and Chivers (1998) delves at work expectations, knowledge and skill input metrics, personal traits, and entrepreneurial characteristics *via* a holistic classification of interrelated job-related skill sets. These skill sets include cognitive, functional, personal, and meta-competencies. On the other hand, researchers also incorporated the aspects of individual entrepreneurial orientation (IEO) in explicating entrepreneurial competency. IEO is a unidimensional construct consisting of proactiveness, innovativeness, and risk-taking (Zmich et al., 2018). This incorporation of IEO is exemplified in the works of Man et al. (2008) and Mitchelmore and Rowley (2010). By incorporating this approach, researchers could focus on specific dynamic competencies cultivated among entrepreneurs by excluding personality traits that are largely stable and difficult to modify (Tittel and Terzidis, 2020). Idealistically, the skills of the entrepreneur change as the venture progresses through its stages of development.

Although a variety of competing models exist, scholars have raised doubt that no single concept alone can significantly predict entrepreneurial success. For instance, Gianesini et al. (2018) compared three mainstream entrepreneurial competency models and concluded that the different domains of entrepreneurial competency possess different levels of specificity and details, making these concepts to be incomparable to one another in terms of superiority and applicability. Furthermore, despite these extant academic studies examining and establishing competency-based frameworks for entrepreneurs, the scope seems to overlap and intertwine with the leadership and managerial disciplines (Mitchelmore and Rowley, 2010; Tittel and Terzidis, 2020). Given these limitations, this research aims to shed some light on reducing this ambiguity.

## Literature and hypotheses

### Entrepreneurial competency and business growth

Within the entrepreneurial literature, scholars generally concurred that a successful business venture is driven by the competence and abilities of the individual entrepreneur (Man et al., 2002; Reis et al., 2020; Riyanti et al., 2022). External market pressures such as shorter product life cycles, cut-throat pricing by aggressive competitors, and regulatory changes are constantly threatening small firms. Individuals who possess innovative and opportunity-seeking abilities would be able to absorb these pressures while growing the business. In addition, being analytical by striking a delicate balance between idea generation and risk-taking allows the owner-managers to exercise *‘street-smart’* and prudent behaviors in the face of market uncertainty and technological turbulence. Moreover, connecting with the right networks allows them to develop mutually beneficial relationships with customers, partners, suppliers, and core stakeholders. In managing the competitive landscape, the effective realization of entrepreneurial competencies should result in productive market-oriented behaviors (Crick, 2021). In tandem with this argument, scholars concurred that entrepreneurial competencies equip the owner-managers to survive or succeed in a competitive business environment. For instance, Al Mamun and Fazal (2018) highlighted that entrepreneurial competency positively influences micro-enterprise firm performance. In a similar vein, entrepreneurial competence contributes to firm performance *via* product innovativeness (Ng et al., 2019). In view of the discourse, this study posits the following hypothesis:

*H1:* Entrepreneurial competency has a positive effect on business growth.

### Entrepreneurial competency and quality of life

The importance of entrepreneurship is gradually transcending beyond traditional academic boundaries, from venture performance to psychological and non-work-related results. Aside from the materialistic appeal, entrepreneurs also possess the intrinsic motivation to embrace life contentment. As human beings, entrepreneurs seek to endeavor challenges to reach terminal values or end goals beyond the sphere of professional success and career recognition. These include happiness, self-respect, equanimity, and leading a prosperous life (Peters and Schuckert, 2014). The autonomy that entrepreneurship provides (e.g., becoming their own bosses, deciding on what hours to work, how much to pay, and when to take vacations) makes quality of life an attractive prospect for initiating a venture. Because small business entrepreneurs aspire to enhance their quality of life, their entrepreneurial behaviors are tailored toward lifelong learning and hard work to achieve success. The impact of entrepreneurship on quality of life has been explored from various perspectives. In terms of communal benefits, higher levels of entrepreneurship have a net positive impact on societal quality of life due to job creation opportunities (Morris and Sexton, 1996). Likewise, study of 24 nations of Woodside et al. (2019) across five continents found that nations highly supportive of nurturing entrepreneurial behavior consistently achieve a higher quality of life scores than nations with lower entrepreneurial behavior configuration scores. In terms of individual satisfaction, entrepreneurial engagements are associated with quality of life attributes, such as freedom, work-life balance, health, and happiness (Peters and Schuckert, 2014; Chakraborty et al., 2019). Drawing upon these facts, we offer the following hypothesis:

*H2:* Entrepreneurial competency has a positive effect on quality of life.

### Entrepreneurial competency and sustainable entrepreneurial intention

Understanding entrepreneurship requires an understanding of entrepreneurial intention, since it reflects one’s desire to own a business (Krueger Jr et al., 2000). Despite the interest in entrepreneurial intentions, there is still only limited evidence about entrepreneurial intentions in different entrepreneurship contexts. Many of these entrepreneurial intention studies are focused on students and prospects with little or no prior business experience. Beyond the entrepreneurial education theme, few studies have adequately explained intentions to remain in an entrepreneurial career (Marshall et al., 2019). In line with the operationalization put forth by Polas et al. (2021), we defined sustainable entrepreneurial intention as the business owner’s intention to sustain in an entrepreneurial career. Not to be confused with sustainability-oriented entrepreneurial intention that factors in environmental consideration (Vuorio et al., 2017), the term “sustainable” refers to an entrepreneur’s propensity to remain in an entrepreneurial career. This connotation also reflects the long-term desire to remain in pursuit of business ownership rather than other forms of employment. The SDT theory is in harmony with sustainable entrepreneurial intention since it elucidates how business owners control their future while remaining professionally and socially competent amid persistent challenges. As small companies are typically under-resourced, obtaining market intelligence while being entrepreneurially focused simultaneously could be too costly to materialize (Hamzah et al., 2023). Due to a lack of resources, incorrect judgments are made, such as pursuing unprofitable markets, taking poorly calculated risks, investing in the wrong products, and making other poor choices (Crick et al., 2021). To stay relevant in the business for the long haul, entrepreneurs need to remain competent to prevent these miscalculations and navigate themselves *via* the correct path. Therefore, the following research hypothesis is offered:

*H3:* Entrepreneurial competency has a positive effect on sustainable entrepreneurial intention.

### Indirect effects of internal locus of control on venturing outcomes *via* entrepreneurial competency

Individuals who possess an internal locus of control believe they have the ability to control their environment (Rotter, 1996). In other terms, it relates to who or what controls an individual’s destiny. Therefore, individuals with an internal locus of control are more likely to assume that their activities influence the rewards or results they receive. Since their conviction in their own talents makes them more proactive and alert to entrepreneurial opportunities, internal locus of control permits owner-managers to effectively search for and discover worthwhile venture prospects (Asante and Affum-Osei, 2019). Across the entrepreneurial literature, internal locus of control has traditionally been used to rationalize entrepreneurial activities (Krueger et al., 2000; Ndofirepi, 2020). In this regard, an internal locus of control plays a decisive role in building individual intention to sustain an entrepreneurial career. Individuals with an internal locus of control believe that they will succeed in entrepreneurship (Baldegger et al., 2017). People who believe in their skills, effort, and abilities, are more likely to harness and enhance their knowledge and abilities when faced with problems and obstacles.

Previous works have associated internal locus of control with opportunity recognition (Asante and Affum-Osei, 2019), career motives (Baldegger et al., 2017), learning from failure, and recovery capabilities (Zhao and Wibowo, 2021). Notably, these studies do not consider entrepreneurial-related competencies and skills in understanding the entrepreneurial intention-locus of control nexus. The question may thus be raised whether an internal locus of control enables the necessary competency that will eventually unlock their intention to sustain an entrepreneurial career. The attribution toward self could be an effect of previously achieved success in starting a venture and should be relatively stable in predicting one’s entrepreneurial abilities (Schjoedt and Shaver, 2012). Therefore, we argue that internal locus of control will lead to one’s sustainable entrepreneurial intention through entrepreneurial competency.

*H4a:* Entrepreneurial competency mediates the effects of internal locus of control on business growth.

*H4b:* Entrepreneurial competency mediates the effects of internal locus of control on quality of life.

*H4c:* Entrepreneurial competency mediates the effects of internal locus of control on sustainable entrepreneurial intention.

### Indirect effects of external locus of control on venturing outcomes *via* entrepreneurial competency

In contrast to those with an internal locus of control, individuals with an external locus of control view growth prospects as being influenced by outside forces. In other words, they heavily rely on support from others to be successful. Individuals with this personality type are susceptible to external attributions of events and situational threats. As a result, they are anxious and skeptical of transforming an opportunity into a profitable endeavor since any effort exerted is perceived of not leading to any meaningful result (Malik et al., 2014). Therefore, such people may be less likely to persist in performing a task. From the entrepreneurial perspective, excessive attributions to external factors will limit entrepreneurs’ willingness to continue running a business. Since they operate in highly unpredictable and dynamic business environments, entrepreneurs are frequently exposed to these fluctuating external conditions, such as unexpected changes in market competition, needs, and regulations. Although these factors are beyond their control, entrepreneurs who lack resilience may be unable to effectively manage their business operations (Hartmann et al., 2022).

The overdependency on external support and luck—the elements that characterize external locus of control-is expected to negatively affect entrepreneurial judgment and actions following adverse events. These events typically include the failure to secure funds or contracts, the sudden exit of business partners, diminishing market demand, and unfavorable regulatory changes, to name a few. Scholars contended that over-reliance on the external locus of control could jeopardize entrepreneurial outcomes if it is not handled wisely, even though recent studies indicated that this counter-productive psychological trait could co-exist together with the internal locus of control (Arkorful and Hilton, 2021; Hoang et al., 2022).

For instance, external locus of control weakens the impact of opportunity recognition on entrepreneurial intention (Hoang et al., 2022). In another study, external locus of control negatively affects opportunity recognition *via* entrepreneurial intention (Asante and Affum-Osei, 2019). Therefore, we contend that external locus of control potentially disrupts entrepreneurial thoughts and actions, as circumventing difficult situations—rather than confronting them, often demoralizes one’s desire to progress forward. Accordingly, we laid forth the following hypothesis:

*H5a:* Entrepreneurial competency mediates the effects of internal locus of control on business growth.

*H5b:* Entrepreneurial competency mediates the effects of internal locus of control on quality of life.

*H5c:* Entrepreneurial competency mediates the effects of internal locus of control on sustainable entrepreneurial intention.

**Figure 1:**
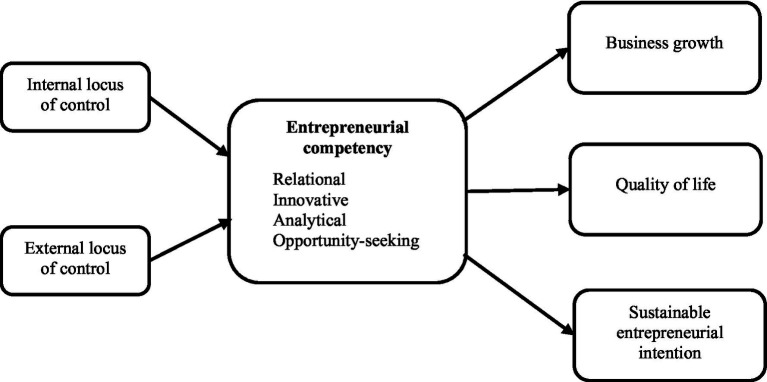
Research framework.

Figure 1 below illustrates our research model, summarizing the hypotheses presented above. The entrepreneurial competency construct is operationalized as a second-order construct with four dimensions: relational, innovativeness, analytical, and opportunity seeking (Man et al., 2008). The construct follows a unidimensional configuration in line with the recommendations of entrepreneurship scholars (see Covin and Slevin, 1989; Zmich et al., 2018).

## Methods

### Samples and data collection procedure

A quantitative research approach was employed to address the research hypotheses, which included the use of a structured questionnaire. We utilized a judgmental/purposive sampling approach to address the study’s research questions. The sample elements were chosen based on their conformity to predefined entrepreneurial-related criteria, despite the nonprobability sampling design and subjectivity of the selection (Botha and Taljaard, 2021). That is, the subject should be comprised of owner-manager of sole-proprietorship or partnership types of businesses that have been operating for at least 2 years.

Furthermore, the selection satisfied the country’s small business criterion of having a revenue of not more than RM15 million (approximately USD 3.43 million) in manufacturing or RM3 million (approximately USD 685,000) in services. Pre-existing directories from SME-related agencies (such as SME Corporation) were used to source the contact details of the entrepreneurs. The survey was emailed and texted to approximately 570 owner-managers, with a realized sample of 165 (representing a low response rate of 28.9%). Sixty-two of the samples were discarded due to missing values, incompleteness, straight-lining responses, and unqualified subjects, hence yielding 102 usable responses.

Although the usable sample is small, it provided reasonable statistical power to test the research model. A power analysis based on the portion of the model with the largest number of predictors was performed using G-Power to assess the sufficiency of the sample size (Cohen, 1988). With two independent variables, the recommended sample size of 68 to obtain a power of 0.80 was exceeded comfortably, assuming a medium effect size of 0.15 and an α of 0.05 (Cohen, 1992). Through the bootstrapping technique, SmartPLS can predict path coefficients of datasets with small sample sizes with the same precision as those with larger sample sizes (Ramayah et al., 2018). Hence, our sample size is deemed adequate, and it is not a severe concern that would jeopardize the results’ integrity. Table 1 summarizes the demographic profiles of the respondents.

**Table 1:** Profiling information on the sampled businesses (*n*=102).

Characteristics		Freq.	%
Gender	Male	57	55.9
	Female	45	44.1
Age	20–30 years old	14	13.7
	31–40 years old	25	24.5
	41–50 years old	42	41.2
	51 years old & above	21	20.6
Sector	Services	32	31.4
	F&B	28	27.5
	Retail & Trading	15	14.7
	Manufacturing	7	6.9
	Others	20	19.6
Business age	2–5 years	50	49.0
	6–10 years	24	23.5
	11–15 years	11	10.8
	16–20 years	7	6.9
	21 years and above	10	9.8
Full-time employees	1–5 people	79	77.5
	6–10 people	5	4.9
	11–15 people	5	4.9
	16–20 people	1	1
	21–25 people	1	1
	>25 people	11	10.8

### Measures

The latent variables were operationalized in the following ways (Table 2 displays a comprehensive list of the multi-item measures and their codes). First, locus of control was measured using an adapted version of scale of Chen et al. (1998). Specifically, internal locus of control and external locus of control were employed as two facets of the locus of control construct; each represented by five items. Most studies throughout the extant psychological literature have suggested that these two forms of locus of control are among the most important aspects of personality since they capture one’s perception of the main underlying causes of events in their lives. Second, entrepreneurial competency was conceptualized as a four-component second-order construct, consisting of relational (six items), innovative (three items), analytical (four items), and opportunity seeking (four items). These items were adapted from Man et al. (2008).

**Table 2:** Confirmatory factor analysis model.

Item	Scale	Loadings	CR	AVE
	**Internal locus of control**			
ILC2	My life is determined by my own actions.	0.703	0.812	0.520
ILC3	I can pretty much determine what will happen in my life.	0.779		
ILC4	When I make plans, I am almost certain to make them work.	0.712		
ILC5	When I get what I want, it’s usually because I worked hard for it.	0.687		
	**External locus of control**			
ELC1	To a great extent my life is controlled by accidental happenings.	0.714	0.818	0.530
ELC3	When I get what I want, it’s usually because I’m lucky.	0.790		
ELC4	It’s not always wise for me to plan too far ahead because many things turn out to be a matter of good or bad fortune.	0.675		
ELC5	Whether or not I get to be a leader depends on whether I’m lucky enough to be in the right place at the right time.	0.727		
	**Ent. Competency (Second-order construct)**			
	Relational	0.771	0.903	0.699
	Innovativeness	0.840		
	Analytical	0.906		
	Opportunity seeking	0.822		
	**Relational**			
REL1	Develop long-term trusting relationships with others.	0.517	0.866	0.525
REL2	Negotiate with others.	0.829		
REL3	Interact with others.	0.854		
REL4	Maintain a personal network of work contacts.	0.631		
REL5	Understand what others mean by their words and actions.	0.685		
REL6	Communicate with others effectively.	0.775		
	**Innovativeness**			
INV1	Look at old problems in new ways.	0.820	0.889	0.727
INV2	Explore new ideas.	0.862		
INV3	Treat new problems as opportunities.	0.875		
	**Analytical**			
AN1	Apply ideas, issues, and observations to alternative contexts.	0.854	0.917	0.736
AN2	Integrate ideas, issues, and observations into more general contexts.	0.909		
AN3	Take reasonable job-related risks.	0.775		
AN4	Monitor progress toward objectives in risky actions.	0.887		
	**Opportunity seeking**			
OP1	Identify goods or services customers want.	0.914	0.940	0.796
OP2	Perceive unmet consumer needs.	0.888		
OP3	Actively look for products or services that provide real benefit to customers.	0.922		
OP4	Seize high-quality business opportunities.	0.843		
	**Business growth**			
BG1	How did the number of employees of the business change over the past year of operation?	0.714	0.937	0.790
BG2	How did the business sales change over the past year of operation?	0.930		
BG3	Has your income from the business increased over the past year?	0.957		
BG4	How did the gross value of the organization’s change over the past year of operation? (Value of assets over liabilities)	0.932		
	**Quality of life**			
QoL1	In most ways my life is close to my ideal.	0.794	0.889	0.618
QoL2	The conditions of my life are excellent.	0.838		
QoL3	I am satisfied with my life.	0.843		
QoL4	So far I have gotten the important things I want in life.	0.793		
QoL5	If I could live my life over, I would change almost nothing.	0.646		
	**Sustainable entrepreneurial intention**			
INT1	I am ready to do anything to sustain my own business.	0.867	0.958	0.852
INT2	I will make every effort to sustain my own business.	0.929		
INT3	I’m determined to sustain my business in the future.	0.963		
INT4	I have very seriously thought about sustaining my business.	0.931		

Third, business growth was measured using a seven-point interval scale with four elements, ranging from 1 to 7, with 1 denoting a reduction of more than 30%, and 7 denoting an increase of more than 30% (adapted from Eijdenberg et al., 2015). Fourth, the quality of life construct was measured using four items adapted from Diener et al. (1985). Fifth, a four-item scale was adapted from Polas et al. (2021) and Vuorio et al. (2017) to measure sustainable entrepreneurial intention. With the exception of business growth, the items for these constructs were measured using a five-point Likert scale that ranged from 1 (strongly disagree) to 7 (strongly agree). Finally, this study controlled for gender, firm age and firm size. In terms of firm size, this variable was measured through the number of full-time employees (Josephson et al., 2016). Two international academic experts who specialized in the theoretical issues and context of this study evaluated and pre-tested the instruments prior to the commencement of the survey.

### Analysis

Structural equation modeling (SEM) is one of the most frequently utilized tools in entrepreneurial behavior research, especially for estimating causal models and hypotheses. SEM allows researchers to test a number of related hypotheses simultaneously by estimating the associations between multiple independent and dependent variables in a structural model (Gefen and Straub, 2005). Partial least square-SEM (PLS-SEM)-rather than covariance-based SEM (CB-SEM), was chosen based on two merits. First, PLS-SEM performs better than CB-SEM in complex models that include latent and hierarchical constructs with a large number of indicators (Chin et al., 2008). Second, PLS-SEM is the preferred approach to maximize the explained variance of the endogenous constructs (Hair et al., 2017). By using the SmartPLS software (Ringle et al., 2015), we tested the measurement and structural models, following Anderson and Gerbing (1988). Four procedures constitute the process of estimating PLS path model parameters. First, an iterative algorithm computes composite scores for each construct; second, attenuation is corrected for the constructs that are treated as factors; third, parameters are estimated; and fourth, inference is tested by bootstrapping (Henseler et al., 2016).

## Results

### Measurement model

The measuring model was evaluated for its reliability and validity. The four standard criteria for evaluating reliability and validity are individual item reliability, construct reliability, convergent validity, and discriminant validity (Hair et al., 2017).

First, since the latent variables are modeled as reflective, the item loadings of the constructs were observed to ascertain their individual item reliability. Majority of the items exhibit loadings higher than 0.7, with the exception of four items with loadings between 0.52 and 0.7. In this regard, although the general rule dictates that item loadings should be higher than the 0.7 threshold, items with lower loadings (0.5 or 0.6) are acceptable as long as the summation of the loadings contributes to average variance extracted values (AVE) scores of greater than 0.5 (Hulland, 1999: Ramayah et al., 2018).

Second, the construct reliability of the main variables was measured *via* the composite reliability (CR) indicator. The CRs for all of the constructs ranged from 0.87 to 0.96, and these figures far exceeded the minimum threshold of 0.7. Third, the AVE was used to assess the convergent validity, and these AVE indicators for all constructs were higher than the 0.5 thresholds. Table 2 presents the values of the loadings, CRs, and AVEs for all of the latent variables. Finally, we assessed the discriminant validity by testing both Fornell and Larcker’s and HTMT criterion.

Based on the Fornell-Larcker’s criterion, the largest squared phi matrix correlation (0.492) was less than the smallest average variance extracted (0.684), signifying no discriminant validity concerns. As for the HTMT criterion, the correlation values are lower than 0.85 and 0.90, according to HTMT.85 (Kline, 2011) and HTMT.90 (Gold et al., 2001) thresholds. Based on these correlation results (Table 3), it can be concluded that the measures did not overlap each other, and discriminant validity is firmly established.

**Table 3:** Discriminant validity.

	ILC	ELC	ENT COMP	BG	QoL	INT
ILC	*0*.*721*	0.298	0.477	0.201	0.355	0.375
ELC	0.182	*0*.*728*	0.256	0.210	0.148	0.138
ENTCOMP	0.395	0.141	*0*.*684*	0.284	0.567	0.447
BG	0.138	−0.163	0.266	*0*.*889*	0.349	0.142
QoL	0.274	0.034	0.492	0.317	*0*.*786*	0.416
INT	0.318	0.008	0.412	0.118	0.374	*0*.*923*

Numbers in italics (diagonal) indicate the square root of the AVE. The figures on the lower left (below the diagonal) reflect Fornell-Larcker’s criterion while figures on the upper right (above the diagonal) follow the HTMT criterion. ILC, internal locus of control; ELC, external locus of control; ENTCOMP, entrepreneurial competency; BG, business growth, QoL, quality of life; and INT, sustainable entrepreneurial intention.

### Structural model

Following the examination of the measurement model, the structural model was evaluated. As a result, the structural model was assessed using the variance explained (*R*^2^) and path coefficient. This study used a bootstrapping approach (5,000 samples) to determine the significance of the path coefficients using *t*-values. These criteria align with suggestions of Hair et al. (2014). The analysis reveals that the structural model explained about 7.1% of the variance in business growth, 24.2% in QoL, 17.0% in sustainable entrepreneurial intention, and 16.1% in entrepreneurial competency.

**Figure 2:**
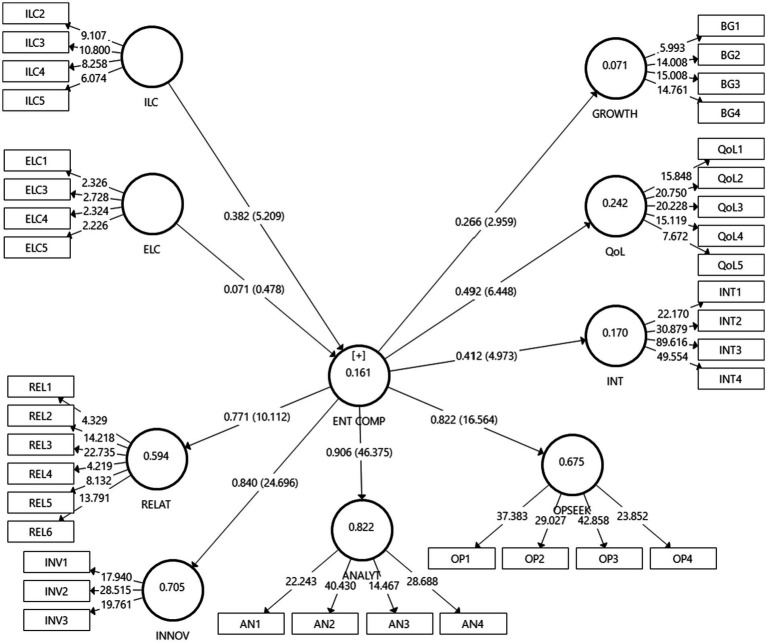
Structural model.

Based on the structural model (Figure 2) and the hypothesis testing (Table 4), six of the nine proposed relationships were significant and supported. First, the hypothesized direct effects were analyzed. The path between ENTCOMP and BG was significant (β = 0.27, *t* = 2.96), fully supporting H1. Similarly, ENTCOMP and QoL’s path was also significant (β = 0.49, *t* = 6.45), confirming the support for H2. Next, the relationship between ENTCOMP and INT was statistically significant (B = 0.41, *t* = 4.97), confirming H3.

**Table 4:** Hypothesis testing.

						Bias corrected		Supported?
No	Path	Coeff.		*t*-value	value of *p*	LLCI	ULCI	
	*Direct paths*							
	ILC → ENTCOMP	0.382	***	5.209	0.000	0.219	0.509	
	ELC → ENTCOMP	0.071		0.478	0.633	−0.409	0.212	
H1	ENTCOMP → BG	0.266	**	2.959	0.003	0.096	0.423	Yes
H2	ENTCOMP → QoL	0.492	***	6.448	0.000	0.315	0.618	Yes
H3	ENTCOMP → INT	0.412	***	4.973	0.000	0.230	0.561	Yes
	*Indirect paths*							
H4a	ILC → ENTCOMP → BG	0.101	*	2.463	0.014	0.036	0.184	Yes
H4b	ILC → ENTCOMP → QoL	0.188	***	3.730	0.000	0.088	0.281	Yes
H4c	ILC → ENTCOMP → INT	0.157	**	3.233	0.001	0.065	0.251	Yes
H5a	ELC → ENTCOMP → BG	0.019		0.425	0.671	−0.135	0.066	No
H5b	ELC → ENTCOMP → QoL	0.035		0.467	0.641	−0.213	0.107	No
H5c	ELC → ENTCOMP → INT	0.029		0.488	0.626	−0.162	0.085	No

Standardized regression coefficients are reported. Bootstrap sample size = 5,000. SE, Standard Error; CI, confidence interval; LL, lower limit; UL, upper limit; ILC, internal locus of control; ELC, external locus of control; ENTCOMP, entrepreneurial competency; BG, business growth; QoL, quality of life; and INT, sustainable entrepreneurial intention. ^*^p < 0.05; ^**^p < 0.01; and ^***^p < 0.001.

Second, the indirect effects for the mediation paths were estimated through bootstrapping procedure (Preacher and Hayes, 2008). We found that ENTCOMP mediated the effect of ILC on BG (β = 0.10, *t* = 2.46, CI = [0.04, 0.18]), the effect of ILC on QoL (β = 0.19, *t* = 3.73, CI = [0.09, 0.28]), and the effect of ILC on INT (β = 0.16, *t* = 3.23, CI = [0.07, 0.25]). Hence, H4a, H4b, and H4c were supported. Unpredictably, ELC was not found to have any indirect effects on BG (β = 0.02, *t* = 0.43, CI = [−0.14, 0.07]), QoL (β = 0.04, *t* = 0.47, CI = [−0.21, 0.11]), and INT, (β = 0.03, *t* = 0.49, CI = [−0.16, 0.09]) *via* ENTCOMP. Thus, H5a, H5b, and H5c were unsupported.

## Discussion

The current study examines the mediation effects of entrepreneurial competency on the link between locus of control on business growth, quality of life, and sustainable entrepreneurial intention. Grounded in self-determination theory (Deci and Ryan, 1985), the exogenous constructs represent an individual’s intrinsic growth inclinations and psychological needs. To recapitulate, this result indicated that entrepreneurial competency directly affects all three entrepreneurial outcomes, namely business growth (H1), quality of life (H2), and sustainable entrepreneurial intention (H3). In addition, internal locus of control, rather than external locus of control, functions as a predictor of entrepreneurial competency. More importantly, our results lend evidence for hypotheses H4a, H4b, and H4c vis-a-vis the mediating pathway of entrepreneurial competency for the links between internal locus of control and entrepreneurial outcomes, namely business growth, quality of life, and sustainable entrepreneurial intention. Contrary to our expectations, external locus of control has insignificant indirect effects on the outcomes *via* entrepreneurial competency; thus, H5a, H5b, and H5c are rejected. Following an empirical survey employing quantitative data from Malaysia, we derive these important contributions to the extant entrepreneurial psychology literature.

First, the nature of relationships among the hypothesized direct paths yielded several key takeaways that narrow the gap within the entrepreneurial psychology literature. These findings also complement and support a few studies examining entrepreneurial competency from the small business setting. For instance, scholars have positively associated entrepreneurial competency with business growth (Al Mamun and Fazal, 2018), and sustainable entrepreneurial intention (Botha and Taljaard, 2021). In justifying the positive causal and effect link between entrepreneurial competency and the outcomes, it is worth noting that entrepreneurship acumen, similar to leadership, is nurtured by commitment rather than inborn genetically or naturally gifted (Biswas, 2022). Owner-managers who equip themselves with the right enterprising roles and skills are in a favorable position to achieve both life and career goals due to their ability to navigate amid resources constraints and hostile environments (Solesvik, 2012). Contrary to the resources-based approach (Barney, 1991) that regards entrepreneurship as a firm value creation process of leveraging resources and assets, entrepreneurship, from the psychological view, emphasizes individuals’ motivation to succeed and exhibit resilience against failure (Zhao and Wibowo, 2021). These individual qualities nurtured over time collectively enable the venture to become equally resilient and progressive.

Second, entrepreneurial competency performs intervention roles in explaining the causal link between internal locus of control and the entrepreneurial outcomes. These outcomes encompass both the career and personal success of entrepreneurs. Although a growing body of studies investigates micro-level entrepreneurial outcomes from the monetary and growth perspectives, personal success, and entrepreneurial sustainability intention received insufficient attention. Our findings imply that positive-thinking entrepreneurs benefit from utilizing their skill sets to achieve a good quality of life and inclination to remain in the entrepreneurial career. On the other hand, having negative attribution and being externally overdependent on others risk the entrepreneur experiencing high levels of task uncertainty and conflicting roles, leading to worsening work satisfaction (Hamwi et al., 2014). The accumulation of this discontent eventually casts doubt on their desire to continue in business. It makes it more difficult for them to imagine what a perfect life accomplishment would be. This phenomenon should explain the absence of any mediating effects involving the external locus of control.

Thirdly, our research demonstrates that entrepreneurial competence is not a significant mediator between external locus of control and entrepreneurial outcomes. External locus of control is also unrelated to entrepreneurial outcomes or competency. To recapitulate, earlier evidence has been very equivocal. External locus of control has a detrimental effect on outcomes including satisfaction (Hamwi et al., 2014) and entrepreneurial intention (Asante and Affum-Osei, 2019). In contrast, exterior locus of control predicts entrepreneurial intentions more strongly than internal locus of control (Hoang et al., 2022; Akorful & Hilton; 2021). Past research suggests that having an internal locus means that a person is self-reliant and self-confident, has strong determination and perseverance, and is most likely to embrace a culture that values individualism and avoids uncertainty (Jain, 2011). This assumption, however, could be comfortably disproven by the fact that Malaysian entrepreneurs, who live in a society with a collectivist and low-uncertainty avoidance culture (Tehseen et al., 2021), rely more on internal than external locus, as the results have shown. Countries with low uncertainty avoidance, for example, may culturally inculcate individuals to be more inclined to take risks and accept ambiguous situations (Hofstede et al., 1997). Consequently, it is plausible that Malaysians do not behave with a locus that is totally devoid of external factors. They may credit positive outcomes to teamwork (due to collectivism) or embrace negative events as pre-destined fate with a silver lining (due to the society’s low levels of uncertainty avoidance).

In a broader sense, entrepreneurial competencies at the micro-level are sometimes misconstrued for fixed, immutable traits based on personal qualities. Entrepreneurial competency neither exists alone nor exists on its own; rather, it is nurtured through positive self-beliefs of own capabilities (Chien-Chi et al., 2020; Wang et al., 2022). We reiterate our earlier stand that skills and abilities—rather than traits (or personal attributes, qualities, and characters) are dynamic and progressive as the entrepreneur gains more maturity and accumulate experience. These competencies are gradually inculcated and remastered by self-reflection and learning from past mistakes (Zhao and Wibowo, 2021). In summary, this study contributes to the literature by resolving some of the intricacies in the realms of entrepreneurial competencies, and professional and life outcomes that consider both internal and external locus of control aspects.

## Implications and limitations

### Practical contributions

The findings offer insight into how entrepreneurs control their psychological traits to develop the necessary competencies for professional and personal success. Therefore, we derive several managerial implications for entrepreneur stakeholders, such as business owners, investors, lawmakers, and public agencies. First, as our empirical findings indicate, owner-managers equipped with the right venturing skillsets and abilities are more likely to experience business continuity, growth, and life satisfaction. The entrepreneur stakeholders, especially public entrepreneurial development agencies, can dedicate the resources that matter most to these entrepreneurs by reinforcing their relationship-building, innovativeness, analytical, and opportunity-seeking skills. Talent development programs that focus on opportunity recognition should enable them to take advantage of promising business ideas while the window of opportunity is still intact (Asante and Affum-Osei, 2019). For instance, competency can be nurtured *via* entrepreneurship competition among youths and university students by educational institutions and public agencies (Wang et al., 2022).

Second, this research demonstrates that if entrepreneurs view life consequences as highly controllable as the results of their individual actions, this attribution should enhance their career and life success *via* entrepreneurial competency enhancement. This research inspires entrepreneurs to instigate a paradigm shift by framing the correct terminal values within their mindset. Stakeholders may instill deeper motivation in entrepreneurs by convincing them that they are the masters of their own destiny. Entrepreneurs should encourage their employees to strive for and exceed benchmarking standards by establishing them in the first place (Biswas, 2022). Besides, entrepreneurs should not discount the opportunity to learn from failure due to involvement in risky actions. Past failures teach them fresh ways to solve problems and limitations and appropriately assess the costs and benefits of each business decision. This retrospection process reinforces beliefs in their own ability and wisdom in undertaking risky activities while actively exploring new ideas, products and markets.

Third, the study may suggest that Malaysians—especially the Malay-ethnic majority, are becoming more independent and less reliant on government-related assistance. Malaysia is among the few countries globally that incorporated an affirmative action policy that guarantees the ethnic Malay majority preferential rights to government projects, public administration recruitment, and tertiary education admissions. Hence, this “*crutch-mentality*” culture, in some ways, goes against the spirit of entrepreneurship by eliminating the psychological aspects of risk-taking and resilience (Shome and Hamidon, 2009). Malaysian entrepreneurs, in some ways as this study has indicated, have gradually shifted their mindset away from the legacy ways of overdependency on government-related assistance and political affiliations. Hence, the law-and policy-makers should reconsider incorporating this positive development into their future entrepreneurial agendas. Money could be well spent on entrepreneurial development rather than outright cash assistance or subsidies. Public funds should be channeled to programs that develop strategic market intelligence and opportunity recognition abilities among youths and potential startups. Besides, entrepreneurs should be encouraged to compete in the open markets rather than chasing government-sourced contracts and procurements.

### Limitations and scope for future research

Similar to other empirical research, this study has some limitations. First, the current model was tested using a cross-sectional survey, which may inflate the chances of common method variance (Podsakoff et al., 2012). A two-wave survey could be used in future studies to analyze the temporal sequence of entrepreneurial competency and outcomes. Second, our study did not offer a balanced view of locus of control expectancy—a combination of internal and external locus of control, also known as *dual control* or *bi-local expectancy* (Torun and April, 2006). External locus of control is not wholly negative in all circumstances. In a challenging business landscape, entrepreneurs with an external locus may assume that their prospects of survival or success are influenced by forces they cannot control, such as market and institutional dynamics. In anticipation of exogenous shocks, a moderate amount of external locus of control may result in greater levels of mindfulness and resilience (Cater et al., 2021; Hartmann et al., 2022).

Third, the challenges brought by the COVID-19 pandemic (e.g., supply chain bottlenecks and operational restrictions) and its subsequent recovery efforts may inflate or deflate the true effect of entrepreneurial competency on business growth. Besides, their perception of government intervention and support programs during the crisis may influence some minor shifts in their locus of control. In this regard, future research can examine the impact of government support and entrepreneurs’ ability to cope with the challenges to their competency, career, and life outcomes. We also encourage researchers to incorporate other relevant and unique variables to add some theoretical values to the existing model, such as entrepreneurial passion (Li et al., 2020), market-oriented behaviors (Crick, 2021), proactive service behavior (Hamzah et al., 2020), and cognitive flexibility (Jiatong et al., 2021), to name a few.

## Data availability statement

The raw data supporting the conclusions of this article will be made available by the authors upon formal request, without undue reservation.

## Ethics statement

Ethical review and approval was not required for the study on human participants in accordance with the local legislation and institutional requirements. The patients/participants provided their written informed consent to participate in this study.

## Author contributions

MH conceptualized the study’s theoretical framework and analyzed the data and wrote the original draft of the manuscript. AO secured the funding *via* a national grant, designed the research methods, facilitated the data collection process, and reviewed and edited the manuscript. All authors contributed to the article and approved the submitted version.

## Funding

The authors would like to thank the Ministry of Higher Education Malaysia for funding this research through the Fundamental Research Grant Scheme [FRGS/1/2019/SS01/UITM/02/15] and the Rector Office, Universiti Teknologi MARA Cawangan Selangor, Kampus Puncak Alam for partially sponsoring this publication.

## Conflict of interest

The authors declare that the research was conducted in the absence of any commercial or financial relationships that could be construed as a potential conflict of interest.

## Publisher’s note

All claims expressed in this article are solely those of the authors and do not necessarily represent those of their affiliated organizations, or those of the publisher, the editors and the reviewers. Any product that may be evaluated in this article, or claim that may be made by its manufacturer, is not guaranteed or endorsed by the publisher.

## Appendix

TABLE A1 Loadings and cross-loadings.

**Table:**

	ILC	ELC	RELAT	INNOV	ANALYT	OPSEEK	BG	QoL	INT
ILC2	**0.703**	0.078	0.183	0.174	0.167	0.306	0.009	0.162	0.186
ILC3	**0.779**	0.094	0.309	0.227	0.309	0.244	0.211	0.274	0.305
ILC4	**0.712**	0.169	0.278	0.198	0.231	0.352	0.106	0.286	0.220
ILC5	**0.687**	0.204	0.165	0.081	0.102	0.280	0.020	−0.028	0.181
ELC1	0.300	**0.714**	−0.013	0.025	0.083	0.225	−0.160	−0.025	0.061
ELC3	0.163	**0.790**	−0.027	0.043	0.122	0.219	−0.043	0.052	0.108
ELC4	−0.003	**0.675**	−0.098	0.080	0.006	0.208	−0.179	0.039	0.017
ELC5	0.026	**0.727**	0.024	0.116	0.047	0.212	−0.131	0.036	−0.146
REL1	0.013	−0.162	**0.517**	0.254	0.345	0.214	−0.020	0.352	0.464
REL2	0.330	−0.114	**0.829**	0.403	0.509	0.332	0.210	0.405	0.235
REL3	0.222	−0.081	**0.854**	0.419	0.523	0.396	0.167	0.429	0.399
REL4	0.270	0.005	**0.631**	0.343	0.401	0.209	0.188	0.287	0.176
REL5	0.264	0.152	**0.685**	0.333	0.387	0.454	0.135	0.292	0.214
REL6	0.323	0.073	**0.775**	0.367	0.372	0.385	0.247	0.439	0.179
INV1	0.135	0.019	0.446	**0.820**	0.654	0.510	0.121	0.280	0.339
INV2	0.207	0.139	0.388	**0.862**	0.673	0.501	0.127	0.249	0.142
INV3	0.292	0.071	0.426	**0.875**	0.688	0.533	0.101	0.232	0.269
AN1	0.320	0.097	0.476	0.614	**0.854**	0.667	0.292	0.432	0.362
AN2	0.251	0.071	0.543	0.720	**0.909**	0.568	0.220	0.479	0.196
AN3	0.272	0.092	0.432	0.630	**0.775**	0.419	0.122	0.419	0.359
AN4	0.189	0.080	0.560	0.737	**0.887**	0.543	0.252	0.406	0.304
OP1	0.380	0.248	0.408	0.519	0.502	**0.914**	0.161	0.222	0.323
OP2	0.279	0.285	0.425	0.469	0.502	**0.888**	0.209	0.241	0.222
OP3	0.355	0.262	0.399	0.536	0.580	**0.922**	0.257	0.268	0.279
OP4	0.432	0.258	0.433	0.617	0.698	**0.843**	0.226	0.325	0.383
BG1	0.179	−0.065	0.173	0.091	0.172	0.111	**0.714**	0.213	0.170
BG2	0.111	−0.120	0.174	0.086	0.177	0.188	**0.930**	0.235	−0.010
BG3	0.165	−0.148	0.219	0.145	0.250	0.288	**0.957**	0.314	0.115
BG4	0.060	−0.215	0.218	0.145	0.301	0.227	**0.932**	0.334	0.140
QoL1	0.241	−0.003	0.411	0.314	0.428	0.204	0.194	**0.794**	0.343
QoL2	0.268	−0.083	0.499	0.173	0.416	0.213	0.393	**0.838**	0.282
QoL3	0.116	0.048	0.356	0.261	0.402	0.313	0.281	**0.843**	0.258
QoL4	0.244	0.014	0.409	0.172	0.351	0.276	0.221	**0.793**	0.410
QoL5	0.210	0.182	0.313	0.247	0.389	0.159	0.136	**0.646**	0.165
INT1	0.370	0.031	0.390	0.283	0.360	0.290	0.152	0.441	**0.867**
INT2	0.246	−0.033	0.303	0.266	0.255	0.233	0.086	0.324	**0.929**
INT3	0.282	0.010	0.373	0.286	0.335	0.346	0.074	0.318	**0.963**
INT4	0.265	0.014	0.301	0.245	0.331	0.376	0.119	0.289	**0.931**

ILC=Internal locus of control; ELC = External locus of control; RELAT = Relational; INNOV=Innovative; ANALYT = Analytical; OPSEEK=Opportunity seeking; ENTCOMP = Entrepreneurial competency; BG = Business growth; QoL = Quality of life; INT = Sustainable entrepreneurial intention.
